# Seasonal abundance of six tephritid species and the fruiting phenology of their main hosts in northern Jiangxi, China

**DOI:** 10.7717/peerj.20751

**Published:** 2026-02-13

**Authors:** Haiyan Yang, Weijun Li, Xin Zhong, Qingxiu Xie, Xiaozhen Li

**Affiliations:** 1 Department of Plant Protection, College of Agronomy, Jiangxi Agricultural University, Nanchang, Jiangxi Province, China; 2 Industrial Development Office, Jiangxi Agricultural University, Nanchang, Jiangxi Province, China

**Keywords:** Tephritid species, Diversity index, Seasonal dynamics, Host fruiting phenology, Northern Jiangxi

## Abstract

Several tephritid species have successfully invaded northern Jiangxi, China, posing a serious threat to regional fruit and vegetable production. This study presents a detailed report on the seasonal abundance of six tephritid species and the fruiting phenology of their main hosts across Nanchang, Yongxiu and Xiushui in northern Jiangxi from 2018 to 2021, using data from methyl eugenol (ME), cuelure (CUE), and sugar-wine-vinegar-water (SWVW)-baited traps and from fruits infested by tephritid larvae. A total of 18,875 tephritid specimens were captured by traps, representing six species, *Bactrocera dorsalis* (Hendel), *B. scutellata* (Hendel), *B. minax* (Enderlein), *Zeugodacus tau* (Walker), *Z. cucurbitae* (Coquillett), and *Callantra trimacula* (Wang). Notably, * B. minax* and * C. trimacula* were discovered for the first time in northern Jiangxi. In addition to *B. minax* (captured only in Xiushui) and *Z. cucurbitae* (captured only in Nanchang), *B. dorsalis*, *B. scutellata*, *Z. tau* and *C. trimacula* were captured in all three regions. *Zeugodacus tau* exhibited the highest population density, followed by *B. dorsalis* and *B. scutellata*, and *C. trimacula* densities were markedly lower. The diversity index of captured tephritid species was similar in Nanchang, Yongxiu and Xiushui, but varied significantly across different seasons from May to December. When tephritid adults were active, * B. minax* population density reached its maximum in mid-June, whereas *B. scutellata*, *Z. tau* and *Z. cucurbitae* peaked in August–September, and *B. dorsalis* peaked in September–October. *Bactrocera dorsalis* and *B. minax* share similar hosts, primarily those from the Rutaceae family, including *Citrus sinensis* (L.) Osbeck, *C. reticulata* Blanco, *C. maxima* (Burm) Merr., and *C. grandis* Tomentosa. However, *B. scutellata*, *Z. tau* and *Z. cucurbitae* have similar hosts, especially those from the Cucurbitaceae family, such as *Cucurbita moschata* (Duch. ex Lam.) Duch.ex Poiret, *Luffa cylindrica* (L.) Roem., *Momordica charantia* L., and *Cucurbita pepo* L. The varying phenological stages of different host fruits guaranteed a continuous and adequate supply of suitable food resources for these tephritid species across different seasons. The theoretical and practical implications of the results concerning the management strategies for the six tephritid species in northern Jiangxi, China, are discussed.

## Introduction

Jiangxi Province is located in southeastern China on the southern bank of the middle and lower Yangtze River, serves as an important production area for fruits and vegetables. The planted area of fruit trees encompasses 438,700 hectares, yielding a total of 7.49 million tons (He et al., 2024), while the planted area of vegetable crops reaches 748,000 hectares, with an output of 18.62 million tons (Li et al., 2024a). The fruit and vegetable sector in Jiangxi Province has emerged as a pivotal cornerstone for driving rural industrial revitalization, making a substantial contribution to boosting farmers’ income levels. Unfortunately, over the past few decades, multiple tephritid species have invaded and successfully established in northern Jiangxi, and thereby posed a considerable threat to the production of fruit and vegetable crops.

Tephritid fruit flies belong to the order Diptera and the family Tephritidae (Duyck, David & Quilici, 2010). The family comprises approximately 500 genera and roughly 4,500 documented species (Hancock & Drew, 2018). Among them, five genera (*Anastrepha* (Schiner, 1868), *Bactrocera* (Macquart, 1835), *Ceratitis* (Macleay, 1829), *Rhagoletis* (Loew, 1862), and *Zeugodacus* (Hendel, 1927) (Wang, 1995; Mitra et al., 2025)) comprising about 50 species (*e.g. A. fraterculus* (Wiedemann), *A. ludens* (Loew), and *A. obliqua* (Macquart) (Díaz-Fleischer et al., 2009), *B. dorsalis* (Hendel), *B. minax* (Enderlein), and *B. oleae* (Gmelin) (Nardi et al., 2005), *C. capitata* (Wiedemann), *C. rosa* Karsch, and *C. catoirii* (Guerin Meneville) (Duyck & Quilici, 2002), *R. pomonella* (Walsh), *R. indifferens* Curran, and *R. zephyria* Snow (Yee et al., 2009), and *Z. caudate* (Fabricius), *Z. cucurbitae* (Coquillett), and *Z. tau* (Walker) (Nair et al., 2017), and others) are particularly notorious for causing significant economic losses in fruit and vegetable production (Clarke et al., 2005). The majority of these economically important tephritid species are predominantly distributed in tropical and subtropical regions, with distinct dominant species occurring in different habitats and geographic zones (Dorji et al., 2006; Leblanc et al., 2015; Cheng & Ye, 2009).

The presence of large tephritid populations can substantially reduce the potential economic value of fruit and vegetable crops. Tephritid fruit flies cause severe direct damage through three interconnected mechanisms: (1) female adults puncture fruit skins with their ovipositors during egg-laying, (2) larvae feed on and destroy internal fruit tissues, and (3) damaged sites become entry points for secondary pathogens, exacerbating crop losses (Li et al., 2019). In areas experiencing severe infestations by tephritid flies, the direct loss of host fruits can range from 30% to as high as 100% (Dhillon et al., 2005). Indirect losses caused by tephritid infestations stem from concurrent reductions in both fruit yield and quality, thereby adversely affecting the marketability of fruit and vegetable crops in domestic and international trade (Vayssières et al., 2015). Tephritid-induced losses vary depending on agro-ecological zones (Vayssières et al., 2008), fruit and vegetable species (Ndiaye et al., 2012), fruit growth stages (Diatta et al., 2013), and crop management practices (Gretchi et al., 2013).

Given their economic significance, the diversity, seasonal abundance, and host range of tephritid fruit flies have garnered considerable research attention. For instance, Kalmath et al. (2023) observed six tephritid species, specifically *B. dorsalis*, *B. correcta*, *B. zonata*, *Z. cucurbitae*, *Dacus ramanii*, and *D. discophorus* through an extensive field investigations conducted from October 2018 to September 2019 within the agro-climatic region of the NorthEastern region of Karnataka. Hasyim, Muryati & Kogel (2008) investigated the seasonal population dynamics of adult male *Z. tau* and quantified its damage to passion fruit orchards in West Sumatra, Indonesia during the 2005 growing season. Vayssières et al. (2015) identified 16 host plant species, such as *Anacardium occidentale* L., *Psidium guajava* L., *Sclerocarya birrea* (A. Rich.) Hochst., *Vitellaria paradoxa* C.F. Gaertn., and *Nauclea latifolia* Smith, near mango orchards in the Sudan zone, noting that these served as critical year-round reservoirs for *B. dorsalis* persistence. These studies have clarified the species composition, occurrence patterns, and damage severity of tephritid fruit flies in those targeted regions, providing critical insights for developing integrated pest management (IPM) strategies. However, in some regions with high tephritid prevalence, biological and ecological data on tephritid fruit flies remain scarce, limiting pest management and conservation efforts.

The coincidence of a warm-humid climate and abundant host fruit availability creates favorable ecological conditions for tephritid occurrence in northern Jiangxi (Liu et al., 2011). To date, four tephritid fruit fly species, *B. dorsalis*, *B. scutellata*, *Z. tau*, and *Z. cucurbitae*, have been confirmed in northern Jiangxi (Li et al., 2024b; Wang et al., 2025). Li et al. (2019) investigated the life history and adult population dynamics of *B. dorsalis* in a commercial citrus orchard in northern Jiangxi from 2008 to 2014, and subsequently developed a seasonally *B. dorsalis* control timeline synchronized with citrus phenological stages. Li et al. (2020) documented the annual population fluctuations of adult *Z. tau* using capture data from CUE-baited traps deployed in a commercial vegetable production area in northern Jiangxi between 2008 and 2015. These studies have laid a robust empirical groundwork for advancing subsequent research on tephritid species, population dynamics, and the associations between tephritids and their host fruits in northern Jiangxi.

This study aims to reveal tephritid fruit fly species in northern Jiangxi, analyze seasonal dynamic patterns in tephritid diversity indices, and investigate their interactions with host fruit phenology. Northern Jiangxi was selected here as our research region due to its extensive cultivaton of tephritid host crops and, more crucially, its role as a key corridor for the northward expansion of tephritid population (Wan et al., 2011). This study differs from previous research conducted in China and other regions. We generated a four-year dataset (2018–2021) through continuous monitoring at six fruit and vegetable production sites in northern Jiangxi, and documented the phenological patterns of 24 host fruit species for six tephritid fly species. We initially confirmed the presence of *B. minax* and *C. trimacula* at multiple fruit and vegetable sites in northern Jiangxi, advanced the understanding of tephritid species succession patterns in northern Jiangxi, and informed the development of integrated, region-specific control strategies for these species. Results may also be extrapolated to other regions in southern China with comparable habitats or environmental conditions.

## Materials and Methods

### Study area

Monitoring of tephritid fruit flies was conducted over four consecutive years (2018–2021) in northern Jiangxi, southeastern China. The monitoring area is located between longitude 114°54′–115°85′E and latitude 28°76′–29°20′N. This area exhibits a distinct four-season climate, characterized by longer summers and winters, and relatively shorter spring and autumn. The annual mean temperature is approximately 17 °C, with summer maxima reaching 42 °C and winter minima dropping to −9 °C. Nearly half of the annual precipitation falls during the April–June period.

We selected three districts (Changbei, Yongxiu and Xiushui) in northern Jiangxi to monitor seasonal fluctuations in multiple tephritid fruit fly species abundance and describe the phenology of their main host fruits. The three districts, Changbei, Yongxiu and Xiushui, were separately equipped with a fruit orchard and a vegetable garden, each covering an area of at least two hectares. These fruit orchards primarily contained Rutaceae species, including *Citrus reticulata* Blanco cv. Kinokuni, *C. maxima* (Burm.) Merr., and *C. sinensis* (L.) Osbeck, served as sites for monitoring *B. dorsalis* and *B. minax*, while these vegetable gardens, where predominantly grew Cucurbitaceae species, such as *Momordica charantia* Linn., *Cucurbita moschata* Duch., *Luffa cylindrica* Roem., and *Cucumis sativus* Linn., served as sites for trapping *B. scutellata*, *Z. tau*, *Z. cucurbitae*, and *Callantra trimacula* (Table 1). There were also various other plants around the fruit and vegetable bases, such as *Arachis hypogaea* L., *Glycine max* (L.) Merr, *Vigna unguiculata* subsp. *sesquipedalis* (L.) Verdc., *Phaseolus vulgaris* L., *Abelmoschus esculentus* (L.) Moench, *Ipomoea batatas* (L.) Lam., *Sesamum indicum* L., and others. These crops received routine maintenance, including weeding, fertilization, and irrigation, as required by their growth stage and environmental conditions. Severe crop diseases and pests were managed through the application of conventional chemical control agents, including insecticides, acaricides, and fungicides. At each experimental site, farmers occasionally removed fruits infested with tephritid larvae, but no species-specific insecticides were applied for tephritid control.

**Table 1:** Information on survey sites for tephritids in northern Jiangxi, China.

Locations	Orchard types	Cultivated crops^a^	GPS coordinates	Altitude (m)	Surface (ha)	Duration	Baits and number of traps^b^	Target species^c^
Changbei	Fruit orchard	B/CC/G/K/L/P_1_/P_2_/RB/T_1_/W_2_	28°76′N, 115°85′E	30	2	2018–2021	ME(16)	Bd
	Vegetable garden	BG_1_/BG_2_/C/CP/E/M/P_4_/TG/WG	28°79′N, 115°83′E	24	4	2018–2021	CUE(17)	Bs, Zt, Zc, Ct
Yongxiu	Fruit orchard	*G*/*K*/*P*_3_/SO/T_1_/TO	29°19′N, 115°82′E	38	∼10	2018–2020	ME(12)	Bd
	Vegetable garden	BG_1_/BG_2_/C/CP/E/RG/WG	29°20′N, 115°76′E	31	6	2018–2020	CUE(12)	Bs, Zt, Zc, Ct
Xiushui	Fruit orchard	CP/P_1_/P_3_/SO/T_1_/W_2_	29°04′N, 114°54′E	155	∼30	2018–2020	ME(12)	Bd
							SWVW(12)	Bm
	Vegetable garden	BG_1_/C/CP/E/P_4_/RG/TG/T_2_/WG/W_1_	29°01′N, 114°62′E	137	3	2018–2020	CUE(12),	Bs, Zt, Zc, Ct

**Notes.**

a B, blueberry, *Vaccinium* spp.; BG_1_, bitter gourd, *Momordica charantia* L.; BG_2_, bottle gourd, *Cucurbita pepo* L.; C, cucumber, *Cucumis sativus* L.; CC, Chinese chestnut, *Castanea mollissima* Blume; CP, chili pepper, *Capsicum annuum* var. *grossum*; E, eggplant, *Solanum melongena* L.; G, grape, *Vitis vinifera* L.; K, kumquat, *Citrus japonica* Thunb.; L, loquat, *Eriobotrya japonica* (Thunb.) Lindl.; P_1_, peach, *Prunus persica* (L.) Batsch.; P_2_, pear, *Pyrus* spp.; P_3_, pomelo, *Citrus maxima* (Burm.) Merr.; P_4_, pumpkin, *Cucurbita moschata* (Duch. ex Lam.) Duch. ex Poiret; RB, red bayberry, *Morella rubra* Lour.; RG, ribbed gourd, *Lagenaria siceraria* (Molina) Standl.; SO, sweet orange, *Citrus sinensis* (L.) Osbeck; T_1_, tangerine, *Citrus reticulata* Blanco; T_2_, tomato, *Lycopersicon esculentum* Mill.; TG, towel gourd, *Luffa aegyptiaca* Mill.; TO, trifoliate orange, *Poncirus trifoliate* L.; W_1_, watermelon, *Citrullus lanatus* (Thunb.) Matsum. et Nakai; W_2_, wonhwang, *Pyrus pyrifolia* (Burm. F.) Nakai; WG, wax gourd, *Benincasa hispida* (Thunb.) Cogn.

b ME, methyl eugenol; CUE, cuelure; SWVW, mixture of brown sugar, wine, vinegar and water. The total number of traps is given in parentheses.

c Bd, *Bactrocrea dorsalis*; Bs, *B. scutellata*; Bm, *B. minax*; Zt, *Zeugodacus tau*; Zc, *Z. cucurbitae*; Ct, *Callantra trimacula*.

### Trapping tephritids

We deployed two trap types, Steiner and McPhail traps, along with three bait types, methyl eugenol (ME), cuelure (CUE), and a sugar-wine-vinegar-water (SWVW) mixture. The Steiner and McPhail traps, and ME and CUE baits were obtained from Jiangxi Entry-Exit Inspection and Quarantine Bureau (Nanchang, China). SWVW was prepared in-house by combining brown sugar, wine, vinegar, and water in a ratio of 1: 1: 4: 30 (w/w), and then added two mL of 45% malathion solution (from Huayu pesticide Co., Ltd, Tianjin, China) to each liter of SWVW to serve as a killing agent. Prior to trap deployment, add three mL of either ME or CUE onto a sponge suspended centrally within the Steiner trap, or pour 300 mL of SWVW into McPhail trap.

Between 2018 and 2021, a total of 93 traps baited with ME, CUE and SWVW were installed within the six sampling sites in northern Jiangxi. Among them, 40 ME-baited Steiner traps were deployed in fruit orchards to capture male *B. dorsalis*, 41 CUE-baited Steiner traps were deployed in vegetable gardens to target males of *B. scutellata*, *Z. tau*, *Z. cucurbitae*, and *C. trimacula*, and 12 SWVW-baited McPhail traps were positioned in fruit orchards in Xiushui specifically to monitor both female and male *B. minax* (Table 1). At each sampling site, we deployed either 3–4 Steiner traps baited with ME, or 3–4 Steiner traps baited with CUE, or three McPhail traps baited with SWVW every year. Traps were deployed annually from May to December, corresponding to the period of adult fly activity (Li et al., 2020).

We carried out comprehensive monitoring of tephritid fruit flies on an annual basis from May to December during the years 2018–2021. All traps were suspended from tree branches or other supports at a height of 1.5–2.0 m above ground level in shaded locations, within easy reach of humans. Traps were placed at a minimum distance of 10 m from the orchard perimeter. To minimize interference, traps were more than 30 m apart from each other, with about four to six traps per hectare. To maintain trapping efficacy, ME and CUE baits were replaced monthly (Wang, 2013), while the SWVW solution was refreshed every 15 days following trap cleaning. Trapped tephritid flies were collected at 6–10 day intervals. If traps were found missing or damaged during monitoring, they were immediately replaced with new ones. Tephritid species captured each time were carefully identified and counted. Subsequently, those identified specimens were classified and preserved in glass bottles containing 95% alcohol, and then stored in the Insect Museum of Jiangxi Agricultural University.

### Host fruiting phenology

To evaluate the host range of each tephritid species, we conducted irregular field surveys in fruit orchards, vegetable gardens, and adjacent areas across Changbei, Yongxiu, and Xiushui districts from May 2019 to December 2021 (Table 1). From these sampling sites, the recently damaged various plant fruits containing tephritid eggs or larvae were collected directly from plants or retrieved from the ground. The key indicator for determining whether a fruit harbors tephritid eggs or larvae is the appearance of brown patches on the fruit’s peel and internal rot of the fruit. In cases of severe infestation, the fruits undergo abscission and drop, while tephritid larvae can be detected within the fruit’s pulp. The quantity, size and maturity of fruits sampled from different crop species were primarily determined by the *in situ* availability of infested specimens. Usually, the fruits of Ebenaceae, Rutaceae, Rosaceae, Solanaceae, and Vitaceae with tephritid eggs or larvae are mature ones. But the fruits from Cucurbitaceae that have tephritid eggs or larvae are mostly young. Concerted efforts were made to ensure the collection of ≥15 fruits per sampling unit, defined by fruit species and location. Infested fruits were stored in ventilated cardboard boxes (40  ×  30  ×  30 cm) and segregated by fruit species. To ensure traceability, each box was labeled with collection date, location, and host information, and then immediately brought back to the laboratory of entomology, Jiangxi Agricultural University.

Each fruit type damaged by tephritid larvae was individually weighed to a precision of 1.0 g using an analytical balance. These damaged fruits were then transferred to sterile circular plastic trays (40 cm diameter × 6 cm height), each lined with a 1.0–1.5 cm layer of disinfected moist sand to serve as the pupation substrate for mature tephritid larvae, covered the top of the trays with fine-mesh gauze nets (10  × 10 threads/cm^2^) to prevent the larvae from escaping. Typically, 2–4 trays were allocated per fruit species for damaged fruits, with each tray containing 400–600 g of fruits, based on the total weight of field-collected samples. Tephritid eggs and larvae within infested fruits were incubated in well-ventilated rooms under ambient environmental conditions for approximately 20 days, until all mature larvae had emerged and pupated. Subsequently, fruit residues were thoroughly removed, and the trays containing tephritid pupae were transferred to an insect-rearing box (78  ×   50  ×   55 cm). The rearing box was supplied with a diet comprising beer yeast, diluted honey (1.0%), and water to nourish newly emerged tephritid adults. Newly emerged adults were reared for about 5 days until they attained full maturity and developed their definitive coloration. During the feeding process, these tephritid species were kept at 27 ± 2 °C with 75 ± 10% relative humidity under natural lighting conditions. After that, these tephritid adults were identified and counted. We assessed tephritid infestation density based on larvae count per kg of damaged fruits: 1–24 larvae/kg indicates low, 25–49 larvae/kg indicates moderate, and ≥50 larvae/kg indicates high infestation density.

### Data analysis

Thirteen trapping data were missing, with seven from Changbei and six from Yongxiu. These missing data were excluded from the analysis of tephritid species diversity and seasonal dynamics. We obtained the yearly catches of a certain tephritid species in a trap by adding up its weekly catches all year round. The yearly catches were aggregated across all traps within a region and years between 2018 and 2021 to obtain the regional total for that species. The yearly catches for all traps were also utilized to compute the average yearly catches, specifically the average captures per trap per year.

Each year’s trapping time was from May to December. Each month in the eight months was partitioned into the first, middle, and last ten-day periods, resulting in a total of 24 ten-day intervals. We used Explore procedures to calculate the means and standard errors of captures per trap per week for each tephritid species within each time interval. Subsequently, these statistical values were employed to analyze their seasonal dynamics.

We adopted four ecological indices, namely richness index, Shannon-Wiener diversity index, Simpson dominance index, and Pielou evenness index, to describe the seasonal diversity features of tephritid distribution in the Changbei, Yongxiu and Xiushui districts across 24 consecutive time intervals. The formulas for calculating these indices were as follows (Ma & Liu, 1994).

(1) Richness index *D* =S/ln(N)
(2) Shannon-Wiener diversity index *H*′=$-{\mathop{\sum }\nolimits }_{i=1}^{\mathrm{S}}{\mathrm{P}}_{i}\mathrm{In}{\mathrm{P}}_{i}$
(3) Simpson dominance index *C* =${\mathop{\sum }\nolimits }_{i=1}^{\mathrm{S}}{ \left( \frac{{\mathrm{N}}_{i}}{\mathrm{N}} \right) }^{2}$
(4) Pielou evenness index *E* =H′/In S

In these formulas, *S* denotes the number of tephritid species; *N* represents the total number of individual species within the community; *P*_*i*_ signifies the proportion of the number of individuals belonging to species *i* to the total number of individuals in the community; N_*i*_ indicates the number of individuals of species *i*. To elaborate the differences in the diversity characteristics of tephritids across seasons from May to December, these ecological indices were subjected to a one-way analysis of variance (ANOVA), followed by Tukey’s honestly significant difference (HSD) post hoc test (*P* = 0.05). All statistical analyses were performed utilizing SPSS version 19.0 (IBM, Armonk, NY, USA).

## Results

### Captured tephritid species

**Figure 1:**
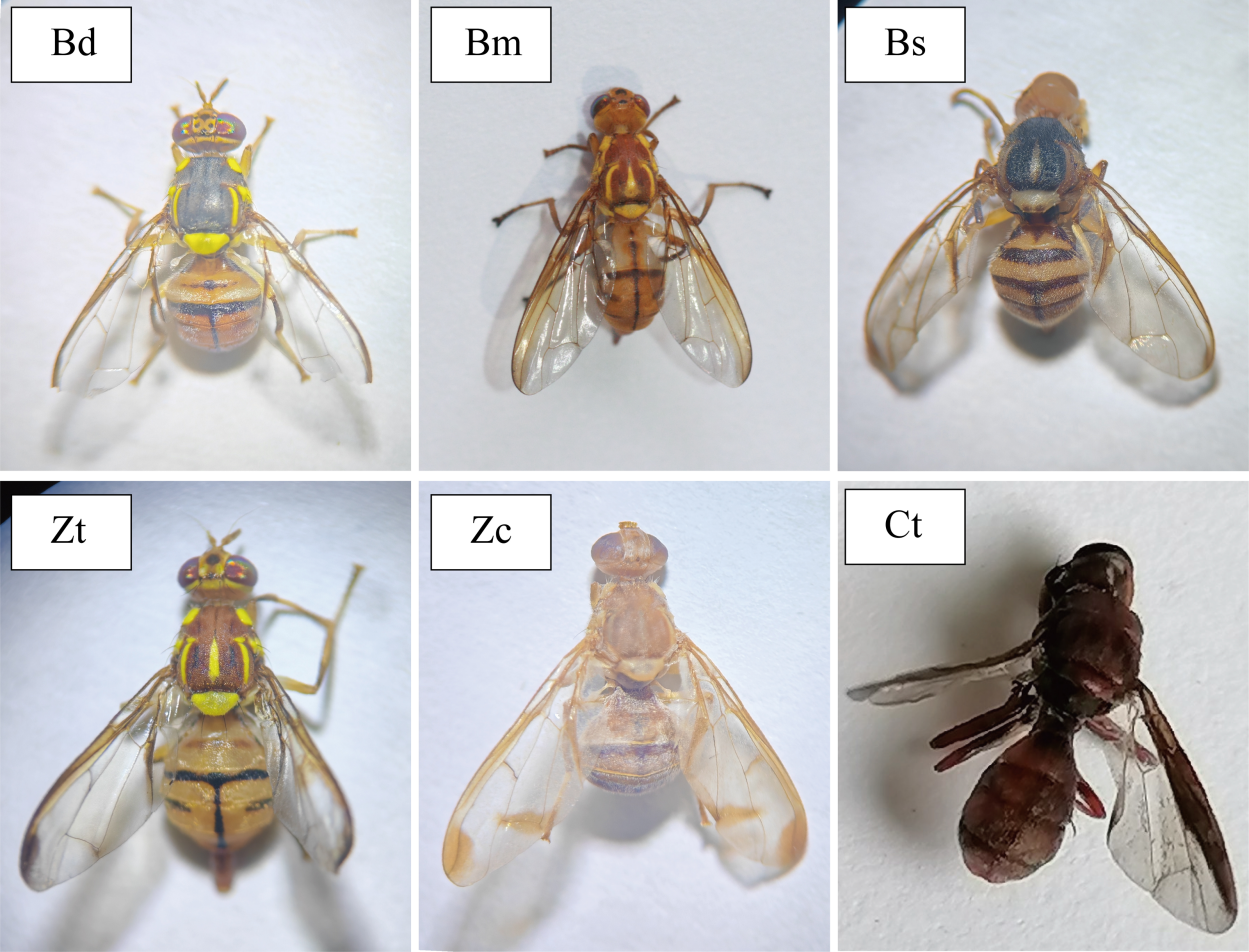
Six tephritid fruit fly species captured in northern Jiangxi, China. Bd, *Bactrocera dorsalis*; Bm, *B. minax*; BS, *B. scutellata*; Zt, *Zeugodacus tau*; Zc, *Z. cucurbitae*; Ct, *Callantra trimacula*.

From 2018 to 2021, a total of 18,875 tephritid fruit fly specimens were collected using ME, CUE, and SWVW baits across 93 traps in northern Jiangxi, China. The collected tephritid specimens belonged to six tephritid species, including *B. dorsalis* (5,257), *B. scutellata* (3,632), *B. minax* (458), *Z. tau* (9,377), *Z. cucurbitae* (47), and *C. trimacula* (104) (Fig. 1 and Table 2). Computed on a per trap per year basis, the captures for these six tephritid species were 131.43 ± 12.33 for *B. dorsalis*, 88.59 ± 7.89 for *B. scutellata*, 38.17 ± 2.60 for *B. minax*, 228.71 ± 14.79 for *Z. tau*, 2.76 ± 0.79 for *Z. cucurbitae*, and 2.54 ± 0.24 for *C. trimacula*, respectively. Notably, *B. minax* and *C. trimacula* were documented in northern Jiangxi for the first time, representing novel regional records (Table 2).

**Table 2:** Species composition and abundance of captured tephritid fruit flies in northern Jiangxi, China between 2018 and 2021.

Species	Regions	Number of traps	Total captures	Percentages (%)	Average captures per trap per year (±SE)
*B. dorsalis*	Changbei	16	3,324	63.23	207.75 ± 18.22
	Yongxiu	12	884	16.82	73.67 ± 1.83
	Xiushui	12	1,049	19.95	87.41 ± 2.38
Total		40	5,257	100	131.43 ± 12.33
*B. scutellata*	Changbei	17	1,992	54.84	117.18 ± 13.79
	Yongxiu	12	1,144	31.50	95.33 ± 5.02
	Xiushui	12	496	13.66	41.33 ± 6.60
Total		41	3,632	100	88.59 ± 7.89
*B. minax*	Xiushui	12	458	100	38.17 ± 2.60
*Z. tau*	Changbei	17	4,348	46.37	255.76 ± 32.41
	Yongxiu	12	2,221	23.69	185.08 ± 11.68
	Xiushui	12	2,808	29.94	234.00 ± 12.05
Total		41	9,377	100	228.71 ± 14.78
*Z. cucurbitae*	Changbei	17	47	100	2.76 ± 0.79
*C. trimacula*	Changbei	17	25	24.04	1.47 ± 0.29
	Yongxiu	12	30	28.85	2.50 ± 0.26
	Xiushui	12	49	47.11	4.08 ± 0.38
Total		41	104	100	2.54 ± 0.24

There were some differences in the average capture of each tephritid species across Changbei, Yongxiu and Xiushui. The captures per trap per year of *B. dorsalis* were 207.75  ± 18.22 in Changbei, 73.67 ± 1.83 in Yongxiu, and 87.41  ± 2.38 in Xiushui. Those of *B. scutellata* were 117.18 ± 13.79 in Changbei, 95.33 ± 5.02 in Yongxiu, and 41.33 ± 6.60 in Xiushui. However, those of *Z. tau* were 255.76 ± 32.41 in Changbei, 185.08 ± 11.68 in Yongxiu, and 234.00 ± 12.05 in Xiushui. No *B. minax* individuals were trapped in Changbei and Yongxiu, and no *Z. cucurbitae* were captured in Yongxiu and Xiushui between 2018 and 2021 (Table 2).

### Abundance indices

The species richness, diversity, dominance, and evenness indices of tephritid fruit flies across different seasons were calculated using sampling data collected from Nanchang, Yongxiu and Xiushui. Table 3 showed that 4–5 tephritid fruit fly species coexisted in each study region throughout the monitoring period. The richness index (*D*) exhibited a range from 0.303 to 1.115 in Nanchang, 0.204 to 1.443 in Yongxiu, and 0.185 to 1.092 in Xiushui. Similarly, the Shannon-Wiener diversity index (*H*′) spanned from 0.562 to 1.417 in Nanchang, 0.425 to 1.305 in Yongxiu, and 0.245 to 1.166 in Xiushui. The Simpson dominance index (*C*) varied between 0.305 and 0.735 in Nanchang, 0.223 and 0.715 in Yongxiu, and 0.124 and 0.697 in Xiushui. The Pielou evenness index ranged from 0.512 to 1.000 in Nanchang, 0.353 to 1.000 in Yongxiu, and 0.353 to 0.994 in Xiushui.

**Table 3:** Abundance indexes of tephritid fruit flies in different seasons in northern Jiangxi, China.

Duration	Changbei					Yongxiu					Xiushui				
	*S*	*D*	*H*′	*C*	*E*	*S*	*D*	*H*′	*C*	*E*	*S*	*D*	*H*′	*C*	*E*
Early May	3	1.116	1.099	0.667	1.000	0	–	–	–	–	0	–	–	–	–
Mid May	3	0.739	1.055	0.640	0.960	2	1.443	0.693	0.500	1.000	2	0.621	0.673	0.480	0.971
Late May	3	0.638	1.097	0.665	0.999	3	1.443	1.040	0.625	0.947	3	0.910	1.061	0.642	0.966
Early Jun.	4	0.851	1.322	0.721	0.954	3	0.805	1.028	0.625	0.936	5	1.092	1.352	0.697	0.840
Mid Jun.	5	1.084	1.417	0.735	0.880	4	1.001	1.305	0.715	0.941	5	0.893	1.151	0.569	0.715
Late Jun.	4	0.681	1.175	0.652	0.848	4	0.874	1.168	0.649	0.843	5	0.726	1.166	0.608	0.724
Early Jul.	5	0.752	0.986	0.539	0.613	4	0.615	0.490	0.223	0.353	5	0.748	1.158	0.618	0.720
Mid Jul.	5	0.690	0.920	0.534	0.572	4	0.491	0.503	0.256	0.363	5	0.657	0.983	0.482	0.611
Late Jul.	5	0.618	0.918	0.537	0.570	4	0.497	0.723	0.429	0.522	5	0.622	1.003	0.500	0.623
Early Aug.	5	0.648	1.009	0.587	0.627	4	0.473	0.843	0.526	0.608	5	0.655	0.990	0.509	0.615
Mid Aug.	5	0.599	0.948	0.550	0.589	4	0.515	0.850	0.535	0.613	4	0.499	0.959	0.550	0.692
Late Aug.	4	0.425	1.057	0.618	0.762	4	0.453	0.947	0.571	0.683	3	0.338	0.860	0.494	0.783
Early Sept.	4	0.442	1.031	0.611	0.744	4	0.543	1.013	0.608	0.731	3	0.349	0.896	0.535	0.816
Mid Sept.	4	0.417	1.083	0.645	0.781	3	0.386	1.026	0.618	0.934	3	0.346	1.036	0.629	0.943
Late Sept.	4	0.458	1.038	0.620	0.749	3	0.344	1.085	0.657	0.988	3	0.334	0.792	0.523	0.721
Early Oct.	4	0.430	1.053	0.629	0.760	3	0.425	0.962	0.591	0.876	3	0.357	0.751	0.465	0.684
Mid Oct.	3	0.321	0.826	0.511	0.752	3	0.382	0.909	0.532	0.827	3	0.363	0.730	0.504	0.664
Late Oct.	3	0.303	0.775	0.483	0.705	3	0.458	0.805	0.497	0.733	2	0.185	0.683	0.490	0.985
Early Nov.	3	0.341	0.562	0.305	0.512	3	0.377	0.566	0.359	0.515	2	0.250	0.689	0.496	0.994
Mid Nov.	3	0.364	0.608	0.370	0.553	2	0.204	0.558	0.371	0.805	2	0.243	0.607	0.416	0.876
Late Nov.	3	0.455	0.905	0.565	0.824	2	0.239	0.425	0.257	0.613	2	0.369	0.245	0.124	0.353
Early Dec.	2	0.481	0.662	0.469	0.955	2	0.455	0.687	0.494	0.991	0	–	–	–	–
Mid Dec.	1	0.000	0.000	0.000	0.000	0	–	–	–	–	0	–	–	–	–
Late Dec.	0	–	–	–	–	0	–	–	–	–	0	–	–	–	–

**Notes.**

S Number of species D Richness index *H*′ Shannon-Wiener diversity index C Simpson dominance index E Pielou evenness index

Seasonal variations were detected in these biodiversity indices. For example, the Shannon-Wiener diversity index (*H*′) in Nanchang were highest in May and June, decreased in July, August, September and October, and reached its lowest values in November and December (*F* = 8.289; *df* = 7, 15; *P* < 0.01). A similar seasonal pattern was observed in Yongxiu (*F* = 5.545; *df* = 7, 15; *P* = 0.031) and Xiushui (*F* = 4.832; *df* = 7, 15; *P* = 0.005). Likewise, the Simpson dominance index (*C*) in Nanchang peacked in May, June and July, then decreased in August and September, and hit its lowest point in October and November (*F* = 4.920; *df* = 7, 15; *P* = 0.005). A comparable seasonal pattern was also noted in Yongxiu (*F* = 12.652; *df* = 7, 15; *P* < 0.01) and Xiushui (*F* = 2.501; *df* = 7, 15; *P* = 0.048).

### Occurrence dynamics

**Figure 2:**
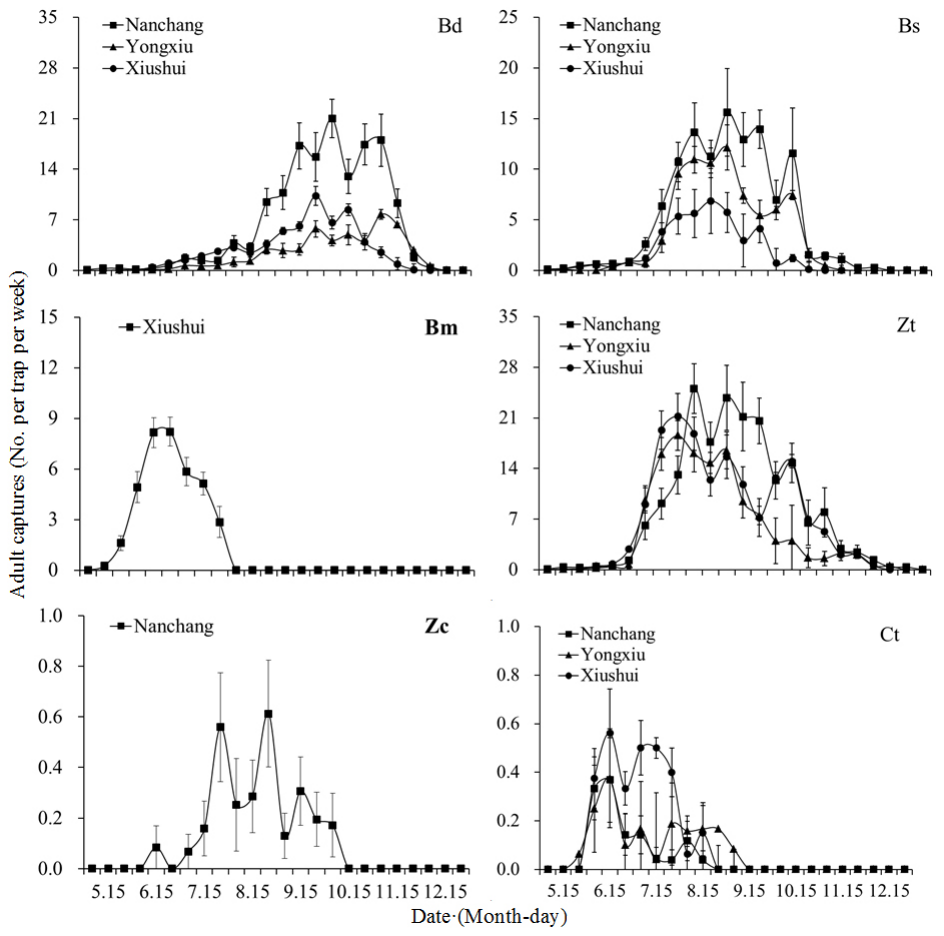
Seasonal dynamics of six tephritid species across three regions in northern Jiangxi, China. Bd, *Bactrocera dorsalis*; Bs, *B. scutellata*; Bm, *B. minax*; Zt, *Z. eugodacus tau*; Zc, *Z. cucurbitae*; Ct, *Callantra trimacula*.

The seasonal dynamics of six tephritid fruit fly populations across Nanchang, Yongxiu and Xiushui were illustrated in Fig. 2. Adult activity periods varied among species: *B. dorsalis*, *B. scutellata*, and *Z. tau* remained active from May to December (8 months annually), whereas *B. minax* was active only from early May to late July (2–3 months annually), and *Z. cucurbitae* and *C. trimacula* from mid-May to mid-October (about 5 months annually). *B. minax* peaked earliest in mid-June, followed by *B. scutellata*, *Z. tau* and *Z. cucurbitae* in August-September, with *B. dorsalis* peaking latest in September-October. During peak abundance periods, the mean numbers of captured adult *B. dorsalis* and *Z. tau* in Nanchang were 21.00 and 25.06 per trap per week, respectively. In contrast, *Z. cucurbitae* and *C. trimacula* exhibited significantly lower abundances, with means of only 0.61 per trap per week in Nanchang and 0.53 per trap per week in Xiushui. During adult occurrence periods, *B. dorsalis*, *B. scutellata* and Z*. tau* exhibited obviously higher densities in Nanchang, whereas *C. trimacula* showed greater density in Xiushui.

### Tephritid occurrence related to fruiting phenology

We found 24 species of host fruits with tephritid eggs or larvae in the fruit and vegetable bases of Nanchang, Yongxiu, and Xiushui. From these infested host fruits, we successfully reared five species of tephritid adults, namely *B. dorsalis*, *B. scutellata*, *B. minax*, *Z. tau*, and *Z. cucurbitae*. But we didn’t obtain *C. trimacula*. Among them, *B. dorsalis* was detected in 10 species of host fruits belonging to the Rutaceae, Rosaceae, Solanaceae, and Vitaceae families. The fruits with the highest infestation density were *Citrus sinensis* and *C. reticulata*, yielding more than 50 larvae per kg of damaged fruits. *Zeugodacus tau* attacked 11 types of host fruits belonging to the Cucurbitaceae and Solanaceae families, with ≥50 larvae per kg of damaged fruits in *Cucumis sativus*, *C. melo*, *Cucurbita moschata*, *C. pepo*, *Benincasa hispida*, *Luffa cylindrica*, *Momordica charantia*, and *Lagenaria siceraria*. *Zeugodacus cucurbitae* was only found in four types of host fruits within the Cucurbitaceae family, and its infestation density was low, with only 1–24 larvae per kg of damaged fruits (Table 4).

**Table 4:** Fruiting phenology and infestation estimation in the main host plants of five tephritid species in northern Jiangxi, China.

No	Fruit species			Fruiting phenology												Tephritid number				
	Common names	Latin names	Families	J	F	M	A	M	J	J	A	S	O	N	D	Bd	Bs	Bm	Zt	Zc
1	Persimmon	*Diospyros kaki* Thunb	Ebenaceae					∗	∗	∗	∗	∗					+			
2	Cucumber	*Cucumis sativus* L.	Cucurbitaceae						∗	∗							+		+++	
3	pumpkin	*Cucurbita moschata* (Duch. ex Lam.) Duch. ex Poiret	Cucurbitaceae						∗	∗	∗	∗					++		+++	+
4	White gourd	*Benincasa hispida* (Thunb.) Cogn	Cucurbitaceae						∗	∗	∗	∗					++		+++	
5	Towel gourd	*Luffa cylindrica* (L.) Roem.	Cucurbitaceae						∗	∗	∗	∗					++		+++	+
6	Bitter gourd	Momordica charantia L.	Cucurbitaceae						∗	∗	∗	∗					+		+++	+
7	Squash	*Cucurbita pepo* L.	Cucurbitaceae						∗	∗	∗	∗					++		+++	+
8	Bottle gourd	*Lagenaria siceraria* (Molina) Standl.	Cucurbitaceae						∗	∗	∗	∗							+++	
9	Muskmelon	*Cucumis melo* L.	Cucurbitaceae						∗	∗	∗								+++	
10	Watermelon	*Citrullus lanatus* (Thunb.) Matsum.	Cucurbitaceae					∗	∗	∗	∗								++	
11	Sweet orange	*Citrus sinensis* (L.) Osbeck	Rutaceae					∗	∗	∗	∗	∗	∗	∗		+++		+		
12	Tangerine	*Citrus reticulata* Blanco	Rutaceae					∗	∗	∗	∗	∗	∗	∗		+++		+		
13	Pummelo	*Citrus maxima* (Burm) Merr.	Rutaceae						∗	∗	∗	∗	∗	∗	∗	+		++		
14	Pummelo	*Citrus grandis* Tomentosa	Rutaceae						∗	∗	∗	∗	∗	∗	∗	+		++		
15	Trifoliate orange	*Poncirus trifoliata* (L.) Raf	Rutaceae						∗	∗	∗	∗						+		
16	Kumquat	*Citrus japonica* Thunb.	Rutaceae						∗	∗	∗	∗	∗	∗	∗			+		
17	Peach	*Amygdalus persica* L.	Rosaceae				∗	∗	∗							+	+			
18	Wonhwang	*Pyrus pyrifolia* Nakai.	Rosaceae					∗	∗	∗	∗					+	+			
19	Loquat	*Eriobotrya japonica* (Thunb.) Lindl.	Rosaceae		∗	∗	∗	∗								+				
20	Eggplant	*Solanum melongena* L.	Solanaceae						∗	∗	∗						++			
21	Tomato	*Lycopersicon esculentum* Miller	Solanaceae					∗	∗	∗						+			+	
22	Sweet pepper	*Capsicum frutescens* L.	Solanaceae					∗	∗	∗	∗	∗				+	++		+	
23	Chili pepper	*Capsicum annuum* L.	Solanaceae					∗	∗	∗	∗	∗					+			
24	Grape	*Vitis vinifera* L.	Vitaceae						∗	∗	∗					+				

**Notes.**

J, F, M, A, M, J, J, A, S, O, N and D represent different months from January to December, respectively.

Bd *B. dorsalis* Bs *B. scutellata* Bm *B. minax* Zt *Z. tau* Zc *Z. cucurbitae*

Number of larvae per kg of damaged fruits: +, 1–24; ++, 25–49; +++, ≥50.

∗ The fruiting periods during which fruit flies cause damage to the corresponding plants.

The fruiting phenology of host plants demonstrated a close correlation with the occurrence patterns of tephritid larvae within fruit and vegetable bases. Among them, *B. dorsalis* larvae feed on host fruits, including *Amygdalus persica* L., *Solanum melongena* L., and *Lycopersicon esculentum* Miller, during the period from May to July each year. After that, they transfer their infestation to various citrus fruits that are cultivated over larger areas. *Bactrocera minax* adult deposit their eggs on *Citrus maxima*, *C. grandis*, and *C. japonica* during the period from June to July. Subsequently, the larvae inflict damage on these host fruits from July to November. *Bactrocera scutellata*, *Z. tau* and *Z. cucurbitae* larvae exhibited overlapping host ranges. They predominantly infested a variety of cucurbit fruits from May to November each year, thereby securing a continuous supply of nutritional resources (Table 4).

## Discussion

The northern area of Jiangxi Province is situated at the northern periphery of the geographical distributions for the majority of tephritid species, serves as a pivotal transitional zone for their northward expansion (Li et al., 2024b). To date, researches on the diversity, geographical distributions, and host plants of tephritid species are primarily concentrated in tropical and subtropical regions (Vayssières et al., 2015; Virgilio et al., 2011). Our research represents the most comprehensive investigation undertaken to date regarding the seasonal abundance of tephritid species and the fruiting phenology of their main hosts in the northern periphery of their distributions. The results deepen our comprehension of the occurrence patterns and range expansion of tephritid species, offering valuable insights for the formulation of region-specific management strategies targeting these tephritid species.

Three different attractants, ME, CUE, and SWVW, were used to catch tephritid adults in the fruit and vegetable farms in northern Jiangxi, China. Among them, ME is a natural phenylpropanoid compound (Heath et al., 2004; Hee & Tan, 2005) that strongly attracts male adults of *B. carambolae*, *B. caryeae*, *B. correcta*, *B. dorsalis*, *B. kandiensis*, *B. occipitalis*, *B. umbrosa*, *B. zonata*, and so on (Tan et al., 2011; Schutze et al., 2014; Zimowska et al., 2024). CUE is highly specific to male adults of *Bactrocera* and *Zeugodacus* species, including *B. cucumis*, *B. scutellata*, *B. tryoni*, *Z. cucurbitae*, *Z. tau*, *Z. vultus*, and so on (IAEA (International Atomic Energy Agency), 2007; Wang et al., 2009). SWVW, a food attractant containing protein hydrolysate and ammonium, attracts female and male adults of multiple tephritid species, especially *B. minax* (Gong et al., 2012). These three attractants demonstrate excellent broad-spectrum properties, effectively trapping multiple tephritid species that are present in south China. Thus, the tephritid species we captured here have encompassed the vast majority of those occurring in northern Jiangxi. Although different attractants were used, the study didn’t compare their trapping efficiency for different tephritid species.

Six tephritid species, namely *B. dorsalis*, *B. scutellata*, *B. minax*, *Z. tau*, *Z. cucurbitae*, and *C. trimacula*, were captured using these three attractants in northern Jiangxi. Among them, *Z. tau* was the dominant tephritid species, accounting for 49.68% of all tephritid individuals captured in all trapping sites. This was further confirmed by the tehphritid-infested fruits collected in northern Jiangxi: the vast majority of tephritids obtained from cucurbitaceous plant fruits that had been damaged by tephritid infestations were identified as *Z. tau* (Table 4). Moreover, cucurbitaceous plants constitute the most widely cultivated crop category in northern Jiangxi. This suggested that the tephritid species captured most frequently were invariably associated with the types and quantities of fruits cultivated locally. Another study by Segura et al. (2006) also confirmed that the extensive cultivation of host plants for *A. fraterculus* and *C. capitata* has resulted in a significant prevalence of these two tephritid species across various fruit and vegetable production areas in Argentina.

It is particularly noteworthy that both *B. minax* and *C. trimacula* have been documented in northern Jiangxi for the first time. Among them, *B. minax* originated in the elevated temperate southern Yunnan-Guizhou Plateau. Afterwards, it spread domestically to Hunan, Hubei, Guizhou, Sichuan, Chongqing, Yunnan, and Guangxi (Tang et al., 2014; Xia et al., 2018), and internationally to countries such as Nepal, India, Bhutan, and Vietnam (Drew et al., 2006; Wang et al., 2014). In northern Jiangxi, we detected *B. minax* adult and larvae solely in a handful of citrus orchards in Xiushui, where it inflicted only a moderate level of damage to citrus fruits (unpublished). The exact time when *B. minax* was introduced into the citrus orchards in Xiushui remains unclear. *Callantra trimacula* has been recorded in four central provinces, including Henan (Mao et al., 2019), Shanxi (Li et al., 2008), Shandong (Shang et al., 2021), and Guizhou (Wang et al., 2016). In this study, *C. trimacula* adult were successfully captured in northern Jiangxi using ME and CUE; however, no *C. trimacula* larvae were obtained from the 24 types of host fruits damaged by tephritid larvae. One plausible explanation was that these plant fruits did not contain eggs or larvae of *C. trimacula*, as they did not serve as host fruits for *C. trimacula*. Another alternative explanation was that the population density of *C. trimacula* in northern Jiangxi was low, with an average of merely 0.55 adults per trap per week during the peak period of adult emergence. The low density ultimately led to the non-detection of *C. trimacula* in these fruits collected. Unfortunately, after reviewing multiple articles on *C. trimacula* (Wang, 1990; Li et al., 2008; Wu et al., 2010), we found no explicit records regarding its host species.

Calculated per trap per year, the capture numbers of six tephritid species were 228.71 (*Z. tau*), 131.43 (*B. dorsalis*), 88.59 (*B. scutellata*), 38.17 (*B. minax*), 2.76 (*Z. cucurbitae*), and 2.54 (*C. trimacula*), respectively. The average capture in northern Jiangxi was significantly lower than those reported in other countries or regions. For example, the catches of *B. dorsalis* could reach 1,600 per trap in June in Chiang Rai, Thailand (Clarke et al., 2001). The combined catches of *B. dorsalis* and *B. zonata* exceeded 885 per trap per month in Gujarat, India (Naganna & Jethva, 2021). A key reason for the lower capture here was that winter temperature limited the increase in tephritid density, as northern Jiangxi was near the northern edge of where most tephritid species live (Li et al., 2024b). Apart from a few pupae, no tephritid larvae or adults were capable of surviving the cold winter. A low survival rate of *B. dorsalis* during overwintering has ever been documented in Wuhan, Hubei, where winter temperature is similar to those in northern Jiangxi (Han et al., 2011).

The richness, diversity, dominance, and evenness indices were used to describe the diversity features of tephritid fruit flies across different seasons in northern Jiangxi. Significant differences were detected in the diversity indices of captured tephritid adults among different seasons. The diversity index peaked from May to June, experienced a slight decrease from July to October, and reached its lowest value from November to December. During May and June, various tephritid species emerge from their overwintering pupal stage into adulthood, with their adult population densities being relatively uniform. This phenomenon substantially elevated the diversity index of tephritid adults during this period. From July to October, there were substantial differences in the types and abundances of available hosts among different tephritid species, leading to considerable variations in the population densities of these species, which impacted the diversity index of tephritid adults during this period. The association between the diversity index of tephritid adults and fruit availability had also been confirmed in mango orchards located in the Borgou Department of Benin (Vayssières et al., 2015). From November to December, due to generational reasons (*B. minax* has only one generation per year) (Dorji et al., 2006) and low population densities (the densities of *Z. cucurbitae* and *C. trimacula* were extremely low), no adults of *B. minax*, *Z. cucurbitae*, and *C. trimacula* were captured, resulting in the lowest diversity index of tephritid adults overall.

For tephritid adults, the peak periods among different species demonstrated considerable variability in northern Jiangxi. Among them, *B. minax* adults reached its density peak in mid-June. This result is credible, given that *B. minax* in south-central China only has one generation each year, with adults occurring from May to July (Xia et al., 2018), and adults exhibit particularly robust vitality in mid-June owing to the optimal ambient temperature (25–28 °C) (Dorji et al., 2006). Moreover, a study conducted by Gong et al. (2012) also confirmed that the density peak period for *B. minax* adults in Chongqing occurred from early to mid-June. *Zeugodacus tau* adults peaked in August-September, and *B. dorsalis* adults peaked in September-October. The factors influencing the peak periods of these tephritid species are multifaceted and complex. Among them, the abundance of available host plants likely played a pivotal role. In northern Jiangxi, the host fruits of *Z. tau*, such as cucumber, pumpkin, bitter gourd and luffa (Allwood et al., 1999; Li et al., 2020), are predominantly abundant from May to September, offering plentiful food resources that contribute to the buildup of *Z. tau* populations. The host fruits, especially citrus (Clarke et al., 2005), become accessible to *B. dorsalis* from August to November, which promote the proliferation and development of *B. dorsalis* populations. The density peak periods of *Z. tau* and *B. dorsalis* are similar to those observed in Hubei and Shanghai (Wan et al., 2011), but different from those in Guangdong, where both *Z. tau* and *B. dorsalis* exhibit two distinct density peaks. Specifically, for *Z. tau*, one peak occurs from January to May, and the other one from October to November; for *B. dorsalis*, one peak is observed in June and July, and the other in September and October (Chen et al., 1995).

Both *B. dorsalis* and *B. minax* larvae infest fruits of Rutaceae plants, but *B. dorsalis* exhibits a stronger preference for tangerines and oranges, whereas *B. minax* primarily targets pomelos (Table 4). Geographically, *B. dorsalis* is mainly distributed in Southeast Asia and some Pacific islands (Schutze et al., 2013; Hu, Wang & Li, 2019); while *B. minax* is concentrated in certain countries or regions within East Asia and Southeast Asia (Dorji et al., 2006). Consequently, these two tephritid species demonstrate minimal ecological niche overlap, resulting in decreased interspecific competition. Compared to *Z. tau*, the population densities of *B. scutellata* and *Z. cucurbitae*, particularly *Z. cucurbitae*, are relatively low. For example, *Z. tau* larvae exhibited densities of ≥50 individuals per kg of damaged fruit in eight host species, while *Z. cucurbitae* larvae were detected on only four hosts, with densities ranging from 1 to 24 individuals per kg of damaged fruit. This may result from interspecific competition, as *B. scutellata*, *Z. tau* and *Z. cucurbitae* share similar host fruits within the Cucurbitaceae family. Moreover, Duyck et al. (2006) demonstrated that intense interspecific competition among the tephritid species, *B. zonata*, *C. capitata*, *Ceratitis catoirii* and *C. rosa*, had caused the population decline of *C. catoirii* in La Reunion, France.

Data collected over a four-year period from six fruit and vegetable production sites in northern Jiangxi provided relevant information for regional tephritid managements. Several integrated management strategies for multiple tephritid species have been developed, including (1) the use of traps baited with ME and CUE, (2) timely removal of infested fruits, (3) fruit bagging to prevent female oviposition, (4) application of GF-120 NF fruit fly bait on cultivated plants (Piñero Jaime, Mau & Vargas, 2009), and (5) biological control using natural enemies such as parasitoids (Satarkar et al., 2009; Vayssières et al., 2009; Vargas et al., 2013). These proven management strategies should be recommended for implementation in fruit and vegetable production regions with high tephritid fruit fly infestation levels. For optimal effectiveness, these interventions should be implemented concurrently at a regional scale and subject to strict oversight by local agricultural authorities.

## Conclusion

The northern region of Jiangxi Province lies at the northern periphery of the geographical ranges where multiple tephritid species are distributed, functioning as a crucial transitional zone for their northward spread. In this region, we identified six tephritid species, *B. dorsalis*, *B. scutellata*, *B. minax*, *Z. tau*, *Z. cucurbitae*, and *C. trimacula*, by using 93 traps baited with ME, CUE, and SWVW. Among them, *B. minax* and *C. trimacula* were recorded for the first time, marking them as new regional records. The Shannon-Wiener diversity index for tephritid fruit flies was highest in May and June, declined in July, August, September, and October, and reached its lowest levels in November and December. The Simpson dominance index peaked in May, June, and July, then decreased in August and September, and hit its lowest point in October and November. *Zeugodacus tau* exhibited the highest population density, followed by *B. dorsalis* and *B. scutellata*, with *B. minax*, *Z. cucurbitae*, and *C. trimacula* displaying comparatively lower population densities. The population density of *B. minax* peaked in mid-June, whereas *B. scutellata*, *Z. tau*, and *Z. cucurbitae* reached their maximum densities from August to September, and *B. dorsalis* attained its peak density from September to October. *Bactrocera dorsalis* and *B. minax* have similar hosts, primarily feeding on the fruits of Rutaceae plants, including *Citrus sinensis* (L.) Osbeck, *C. reticulata* Blanco, *C. maxima* (Burm) Merr., and *C. grandis* Tomentosa; while *B. scutellata*, *Z. tau* and *Z. cucurbitae* exhibited similar host ranges, particularly targeting fruits from the Cucurbitaceae family, such as *Cucurbita moschata* (Duch. ex Lam.) Duch. ex Poiret, *Luffa cylindrica* (L.) Roem., *Momordica charantia* L., and *Cucurbita pepo* L. These findings held significant importance, both for the prevention and control of these tephritid species and for implementing quarantine measures in areas where they are not currently distributed.

##  Supplemental Information

10.7717/peerj.20751/supp-1 Supplemental Information 1 Monitoring data

10.7717/peerj.20751/supp-2 Supplemental Information 2 Rearing data

## Additional Information and Declarations

### Data Availability

The following information was supplied regarding data availability: The raw data is available in the Supplemental Files.
